# Ten simple rules for developing a mentor–mentee expectations document

**DOI:** 10.1371/journal.pcbi.1005709

**Published:** 2017-09-21

**Authors:** Kristyn S. Masters, Pamela K. Kreeger

**Affiliations:** 1 Department of Biomedical Engineering, University of Wisconsin-Madison, Madison, WI, United States of America; 2 Carbone Cancer Center, University of Wisconsin School of Medicine and Public Health, Madison, WI, United States of America; 3 Department of Medicine, University of Wisconsin School of Medicine and Public Health, Madison, WI, United States of America; 4 Department of Cell and Regenerative Biology, University of Wisconsin School of Medicine and Public Health, Madison, WI, United States of America; Whitehead Institute, UNITED STATES

## Introduction

There is general agreement that effective mentoring is beneficial for mentees, mentors, and overall scientific productivity [1, 2]. Discussions of what to consider in mentoring philosophies and mentor–mentee relationships have been published [3–5], and discipline-specific versions of a curriculum to develop mentoring skills are available (https://mentoringresources.ictr.wisc.edu). However, these resources focus on general concepts about mentoring, such as the importance of communication, consistency, and accessibility. In contrast, concrete strategies to improve the mentor–mentee relationship have been more difficult to define [6]. Funding agencies such as the National Science Foundation (NSF) and National Institutes of Health (NIH) have supported the implementation of policies aimed at improving this relationship. For example, in 2009, the NSF began requiring the inclusion of mentoring and development plans in grant proposals that request support for postdoctoral fellows; similarly, in 2014, the NIH announced (NOT-OD-14-113) that annual grant progress reports would be required to describe whether and how individual development plans (IDPs) are used to manage the career development of predoctoral and postdoctoral trainees. While a development plan can be effective as a tool to help a mentor and mentee work towards the mentee’s long-term goals, this document alone is insufficient as it does not address the day-to-day operations of the lab—the source of many conflicts for both mentors and mentees.

Another resource that provides guidance on developing positive mentoring relationships is the “Compact Between Postdoctoral Appointees and Their Mentors” released by the Association of American Medical Colleges (AAMC) [7, 8]. Included in this compact are general principles governing responsibility for career development, development of the research plan, the need for regular feedback, and ethical conduct. However, this society-level document is lacking in details that would outline how these specific guidelines will be followed. Therefore, we have found it effective to develop “expectations documents”—lab-specific documents that detail both big picture elements of the mentor–mentee relationship as well as some of the nitty-gritty rules of how the lab operates. By clarifying the norms for a particular lab, an expectations document can provide a mechanism for prospective mentees to evaluate if a lab will be a good match for their needs. So, how do you develop your own expectations document? See our 10 simple rules below!

## Rule 1: Write it down

This may seem obvious, but it is important to remember that a written document more clearly and consistently communicates your expectations than conversations. Written documents also allow for both mentor and mentee to revisit the expectations as the mentoring relationship develops and provide documentation should a situation arise where either mentor or mentee does not adhere to the predetermined expectations. While it can be challenging to construct the initial document, even an incomplete draft could offset major clashes between mentor and mentee. To get started, you may be able to get a template from your institution, department, or training program, which will incorporate university policies and procedures. Alternatively, to help you as you start this process, we have included a sample expectations document (Suppl. File 1). This document is a modified version of the documents we utilize with our graduate student trainees. Which brings us to Rule 2.

## Rule 2: Tailor the expectations document to your audience and environment

In most research labs, there are personnel at a variety of career stages—postdocs, graduate students, undergraduates, and other scientific staff. Each of these groups has unique needs to address; as a result, it is useful to have separate documents for different personnel groups. Examples of graduate specific elements in the provided sample include indicating that the student is responsible for fulfilling course requirements, but that the mentor is available to help guide these decisions. For undergraduates, you may choose to discuss your grading policy, while for staff you may discuss their role in lab management, and the version for postdoctoral researchers may emphasize expectations regarding leadership and independence. You will also want to tailor your document to the type of research environment that your mentees work in. As discussed in a later rule, expectations may differ for research settings that are theoretical, computational, experimental, fieldwork based, or a combination of these environments.

## Rule 3: Convey the big picture

Ideally, the expectations document should provide the mentee with an understanding of your lab culture and approach to their training. Providing an overview of the lab environment as well as describing your mentoring philosophy can assist the mentee in establishing a positive relationship with both you and the other lab personnel. This information can also help prospective mentees determine whether your lab is an environment where they can picture themselves thriving. In our example, we provide both an overview paragraph summarizing these elements as well as comments throughout that relate our mentoring philosophy.

## Rule 4: But don’t forget the nitty gritty

At this point, you may be wondering if it would be easier to use the published mentoring guidelines from the AAMC [7, 8]. While these guidelines provide an excellent source for developing your big picture philosophy, in our experience it has been beneficial to move beyond the mentoring philosophy and also convey some of the specific rules of the lab. It is not feasible to concisely list all guidelines related to lab performance or work expectations—however, clearly stating these rules can prevent significant conflict in the mentoring relationship. In our example document, we discuss hours and vacation, detail the overall requirements for lab safety and lab jobs (leaving further specifics to our lab protocols), conflict resolution, and outline how authorship is determined. For research that is theoretical and/or computational, it may be important to discuss policies on working remotely and documentation requirements for codes, while for fieldwork, discussion of expectations related to conduct and safety would be appropriate. Ultimately, you will want to confirm that the expectations that you outline for your mentees are consistent with the rules and regulations of your institution.

## Rule 5: Expectations are a two-way street

Just as you will outline your expectations for the mentee’s behavior, it is important to outline what they can expect from you. Mentoring styles differ, and alignment between mentoring style and a mentee’s self-identified needs can benefit both parties. For example, a student who wants regular feedback may struggle while working with a mentor who prefers a hands-off approach.

## Rule 6: Articulate boundaries

When constructing your expectations, be mindful of the power differential that exists between you and your mentee. The expectations document may be used to communicate professional boundaries, such as whether the mentee will be expected to contribute to work commonly performed by the mentor (e.g., our example includes discussion of assistance with grant preparation, advising other group members). Additionally, you can use the expectations document to articulate personal boundaries. For example, to maintain work–life balance, we have included information in our example on how much time a student should expect for answers to their questions and situations where it would be appropriate for the mentee to call on a personal number.

## Rule 7: Work with others to develop your expectations document

Are you feeling stuck or overwhelmed? Getting input from people with different perspectives may make it easier for you to develop your expectations document and determine sections that need more detail or clarification. For example, you may want to discuss your document with your own mentors, colleagues, or your more senior mentees. One especially effective strategy is to develop a small writing group with a few colleagues where each member develops an expectations document over the span of several meetings. In addition to their insights, the peer pressure to have a completed document for the next meeting may help to motivate you to complete this task.

## Rule 8: Plagiarism is okay (sort of)

As you look through examples or work with your colleagues, it is likely you will find statements that resonate with your approach. Because one aim of drafting an expectations document is to simplify the job of being a mentor, we would encourage you to ask for permission to copy and/or modify existing statements. Consistent with this, we grant permission for you to copy and/or modify sections of the example expectations document (Suppl. File 1). However, we encourage you to think critically and be certain that any statement that you use truly reflects your actual mentoring approaches—this is essential to prevent sending mixed messages to your mentees.

## Rule 9: Encourage regular conversation about the expectations document’s interpretation

When first starting in a lab, a mentee’s understanding of the expectations document will be largely theoretical. However, as the mentee progresses through their training and sees the mentoring expectations put into practice, new questions may arise regarding the interpretation and implementation of these guidelines. Regular conversations about the expectations document can help maintain an open channel of communication, head off misunderstandings, and provide feedback for document revision. In addition to informal conversations, it may be beneficial to set aside a part of one group meeting each year for this or incorporate it into your lab’s evaluation process. These conversations lead to our final rule.

## Rule 10: This is a living document

As noted in Rule 1, it is appropriate to start with a smaller expectations document and add or refine content over time as needed. Even for those who start with a complete expectations document, unforeseen situations will arise. In addition, the rules of the graduate program or institution may change over time. Regular revisions to the expectations document allow for these changes in expectations to be incorporated so that all members of the lab remain on the same page.

## Conclusions

Like any other type of relationship in a person’s life, the relationship between a mentor and a mentee requires intentional effort and clear communication to be healthy and successful. Providing your mentees with a guiding document about the expectations in your research lab benefits all parties. Sharing this expectations document with prospective lab employees can help them assess whether your lab is an environment that is likely to meet their needs and help you avoid a hiring mismatch. Once a mentee has joined your lab, the presence of written expectations can reduce the potential for conflicts and misunderstandings, which are damaging to the productivity and happiness of both the mentor and mentee. We hope that these 10 simple rules help you to develop an expectations document that works for your lab in order to lessen conflict and improve productivity.

## Supporting information

S1 File A sample mentor–mentee expectations document. (DOCX)
