# Supplementary material for: Ten simple rules for developing a mentor–mentee expectations document
Source: PLoS Comput Biol. 2017 Sep 21;13(9):e1005709. doi: 10.1371/journal.pcbi.1005709 (PMC5608163; doi:10.1371/journal.pcbi.1005709)
Supplement: S1 File — (DOCX) [file pcbi.1005709.s001.docx]

**Welcome to the Lab!**

**Mission Statement**

My lab has three central goals:

- To do quality science
- To develop each lab member to become a successful scientist
- To maintain a collegial and intellectually-stimulating environment

As your Ph.D. advisor, I will provide the mentorship and training needed to help you develop into an independent scientist. To accomplish this goal, it is important that we establish effective communication and align expectations with each other. This document provides a framework for communicating the culture of my lab, and how you and I will work together to further your scientific productivity and intellectual development. I believe in mentoring each individual in a manner that best meets their needs, and I look forward to having open discussions about these expectations and revisiting them as necessary to enable your successful professional development. Please note that this document is not a substitute for university rules and regulations, and that those policies and any legal requirements supersede anything in this document.

**What you can expect from me**

- **I will set the scientific direction for the lab and provide the means to pursue those directions**. This will include helping you to find a research topic, writing grants to fund the research, and maintaining the necessary university protocols for us to utilize the laboratory. Additionally, I will seek out collaborators for our work to further your opportunities.
- **I am committed to mentoring you now and in the future.** I am committed to your education and training while in my lab, and to advising and guiding your career development. I will work to promote you and your work.
- **I will encourage you to attend scientific meetings and make an effort to fund these activities.** These meetings are important to showcase your work and for the networking opportunities as you pursue positions after your time in my lab ends.
- **I will be available for regular meetings and will provide timely review of research.** In addition, I will do my best to provide an open door policy and respond quickly to e-mails. Please be aware that there will be times when I will be unavailable due to other obligations. For abstracts and small data questions, I will generally be able to review in 1-2 days, for papers and thesis, I will need 1-2 weeks. In the event of a lab emergency, I may be contacted on my cell phone.
- **I will provide a work environment that is intellectually stimulating, supportive, safe, and free from harassment.** I take seriously any difficulties you experience in relationship to this statement – if there are conflicts with another lab member, please inform me and I will work with you and the other lab member to find a resolution. I will strive to understand your unique situation and am open to your suggestions on how to improve your experience in the lab.

**What I expect from you**

***You will take ownership of your educational experience***

- You will need to **determine the requirements for your individual graduate program and are responsible for insuring that you are in compliance**. As you progress, I will work with you to select courses, qualifying exams, and committee members.
- You will **keep me updated on your research progress and challenges.**
- To earn your degree **you must transition towards independence**. We will work together to track this process, but ultimately when you earn a degree will be up to the work you produce, not simply the time you put in.
- **Seek out professional development opportunities** – being a successful scientist involves more than being good at the bench. You must communicate well (presentations, papers, grants), develop personal skills (lab management, mentoring), maintain high ethical standards, and for a faculty career, teach. However, these opportunities must be balanced with the most important element of your career development – research progress towards your thesis.

***You will develop your personal research skills***

- Begin **reading the scientific literature** - read the papers I suggest, run a literature search and read papers suggested by this search. Spend some time each week updating your literature and just browsing. Subscribe to relevant eTOCs.
- Learn how to **plan your experiments** so that they help you progress on the overall goal of your project. Make sure your experiments address the question of interest correctly – this includes learning how to do the appropriate controls, techniques, etc. You will also need to learn how to effectively plan and multi-task to prevent down times. Develop plans with short/medium/long-term goals.
- Keep **detailed lab notebooks** – these are essential to turn your hard work into a finished paper or thesis. Your notes should allow your work to be reproduced (meaning they must be understandable by people other than yourself) and will help to assign credit for authorship. They are required by funding agencies and for any potential patents. You are required to leave the originals behind when you leave the lab for others to build upon your work.
- Develop your **writing and presentation skills**. As you start to make progress, begin outlining a paper’s figures and drafting the text. Be prepared to go through rounds of revisions before submitting an abstract or paper. Although the availability of travel funds will vary, I encourage you to submit your work for presentation at one conference per year. **Attend relevant seminars** – I suggest 1-2/month to learn both science and how to give a good talk.
- Develop your **mentoring and management skills**. Mentoring undergraduate researchers not only helps you achieve your experimental goals, but also provides an opportunity to further your professional development as a supervisor. As the direct supervisor of an undergraduate student, you will be expected to train them appropriately, provide them with experimental guidance, and ensure that they operate in a safe and respectful manner in the lab.
- Consider **applying for fellowships, traineeships, and travel grants**. Not only will an award help your career and the overall lab funding situation, the experience of writing the proposal will help you think about what you are doing more deeply.
- Learn how to **accept and utilize constructive criticism**. The feedback from me, colleagues, committee members, and course instructors is intended to improve your work.

***You will contribute to the lab and be a good lab citizen***

- Senior graduate students are responsible for helping to train new students in the ways of the world (*i.e.* lab procedures, how individual/group meetings work, literature searching, etc.). **Science is a community** - many people will help you along the way and you should return the favor. Share your insider knowledge of techniques with others.
- **Data belongs to the lab, not to any one individual** – as a result, you will be expected to leave your original notebooks and files when you leave the lab. In addition, there will be times when you will be asked to assist me in submitting grant applications to NIH/NSF/etc. This activity is essential to provide continuing support for the lab.
- You will have **designated lab jobs** such as ordering, general maintenance, taking care of one of the instruments, etc. Failure to do your lab jobs not only affects you, it can impede the entire lab and will not be tolerated. Everyone is expected to help with dishes, making sure that supplies do not run out, aliquotting, reporting problems with equipment to the person in charge, and general lab cleanliness.
- **You will work safely in the lab**. Before beginning in the lab you must complete safety training and will be expected to renew that training as needed. You will follow all safety procedures defined in our lab protocols and immediately communicate any safety concerns to me.
- You will **keep lab protocols** **up-to-date** on the main lab computer.
- When working in the labs of other investigators, be polite, neat, and gracious. Always follow their rules. If something breaks during your use, report it immediately to the appropriate person.
- **Be respectful, tolerant of, and work collegially with laboratory colleagues: respect individual differences in values, personalities, and work styles.**

**Nuts and Bolts**

***Hours and Vacation***

I do not believe in tracking hours – instead, I am interested to see that you are productive. However, if I sense that this is being taken advantage of, the situation will be addressed. You will quickly recognize that biology is not a 9-5 proposition - night and weekend hours come with the territory. I ask that you discuss with me at least 4 weeks before a planned absence - this way we can determine if it is an appropriate time for a vacation and if there are grant or other deadlines during that period we have ample time to prepare. I expect you to satisfactorily complete all assigned research duties prior to your planned departure.

***Meetings***

Come prepared to discuss/present your recent research and next steps. A written agenda including what you have done and what you propose to do in the next week must be e-mailed to me by 3 pm the day before the meeting. You must bring your lab notebook to each meeting.

***Annual Evaluations***

Each year we will have an evaluation – this will help us to determine things that are going well or are areas for improvement. I will tell you if I am satisfied with your progress and help identify steps you can take to fix any concerns. This is also an opportunity for you to communicate to me what I can do to help you succeed. Tell me if you feel that you need more guidance, more independence, to meet more often, etc.

***Authorship***

One of the most important tasks in science is disseminating your research through publications and presentations; therefore, authorship on these items is an important indicator to the outside world of your role. Authorship implies a significant contribution to a paper such as intellectual ideas that change the research or experimental contributions (just following instructions and not actively participating in the experimental design/interpretation will be acknowledged, but likely would not result in an authorship). While the order of authors varies by specific field, in general for bioengineering the first author is the student/post-doc who took the lead and wrote the paper, the last author is the PI, and the authors in between are in order of decreasing contribution. Failure to complete papers before leaving the lab may result in a junior member doing so as the 1^st^ author in your place.

***Conflict Resolution***

If a conflict arises with another lab member during your time in my lab, I will work with you to find a resolution. If the conflict fails to be resolved or you do not feel comfortable involving me, I encourage you to consult with the Department Chair, Chair of your graduate program, or the university Ombuds office to attempt to settle the disagreement.
